# Malaria

**Published:** 1900-12-22

**Authors:** 


### MALARIA.

The last meeting of the Royal Medical and Chirurgical Society was devoted to what the authorities of that institution chose to announce as a discussion on malaria. Really the entertainment consisted of an address by Dr. Patrick Manson and a paper by Dr. Sambon. Although, however, all trace of anything in the shape of a discussion was entirely absent, the meeting was interesting enough to those who wished for a more or less popular repetition of the thrice-told tale of the malaria parasite. The chief points of importance in Dr. Manson's address, outside the interest which always must attach to the general story, lay in what he said as to the gaps in our knowledge. Two things, he said, were required to complete our knowledge of the entire life history of the malaria parasite, namely, first what circumstances determined the direction in which the spore in the human blood should develop—whether it should take on that form which led to the so-called Golgi cycle—multiplication as a blood parasite within the human body with the periodical discharge of spores into the blood stream—or whether it should take on the crescent or bisexual form in which the parasites became capable, when swallowed by the mosquito, of undergoing the "mosquito phase" of the life of the organism. The second point in regard to which we were ignorant, he said, was as to what became of the parasite during the long latent period which sometimes elapsed between the exposure to infection and the attack. A man might, he said, receive the infection in India or some malarious country, and at the time show no sign of the disease, no shivering, no malaise, no anything; and then perhaps a couple of years afterwards, he might be taken ill with malaria, say, while walking down Regent Street, and we badly wanted to know what happened to the parasite to cause it to remain latent in the patient's body all this time, and what happened as he walked down Regent Street to suddenly rouse it into activity. Turning then to the question of blackwater fever, Dr. Manson's criticism was chiefly destructive of the theories put forward by various observers, Koch among the rest. He did, however, make a suggestion which is worth bearing in mind—namely, that possibly blackwater fever might be an entirely independent disease, due to an infection *sui generis*, and that its undoubted connection with malaria might be due to the parasite of blackwater fever not developing or leading to symptoms except in the presence of a coincident malarial infection, and he showed by analogy that such an association did exist in regard to some organisms. This, however, was but an hypothesis. Finally, he pointed out that although no one of the various means which we now possess of combating the infection of malaria is by itself universally trustworthy in the ordinary conditions of life, and that although complete protection is perhaps impracticable, yet a relative degree of protection is always attainable and is of immense value. Dr. Sambon then, speaking for himself and Dr. Low, described the details of the well-known experiment of the mosquito-proof hut, of which they had been the heroes, and that of the transit of the infection by specifically infected mosquitos from Rome to London, in the carrying out of which Mr. Thorburn Manson had allowed himself to be bitten by the infected mosquitos, and by then developing malarial disease of the same type as that of the patient in the Roman hospital on whom the mosquitos had fed a few days before, had clinched the proof that mosquitos were the agents by which the disease was carried. All of which was very interesting—but it was not what is generally understood by a discussion.

